# First Report of *Criconema demani* from Russia

**DOI:** 10.21307/jofnem-2019-019

**Published:** 2019-04-17

**Authors:** Sergei Tabolin, Irina Markina

**Affiliations:** 1 Center of Parasitology, A.N. Severtsov Institute of Ecology and Evolution, Russian Academy of Sciences, Moscow, 119071, Russia; 2 All-Russian Scientific Research Institute of Fundamental and Applied Parasitology of Animals and Plants named after K.I. Skryabin, Russian Academy of Sciences, Moscow, 117218, Russia

**Keywords:** *Criconema demani*, Criconematidae, Molecular characterization, Morphology, Nematode, *Rubus idaeus*, Russia, Taxonomy

## Abstract

A population of the criconematid species, identified as *Criconema demani* (Micoletzky, 1925), is reported from a natural habitat in northwestern Russia. Measurements and morpho-anatomy obtained with light microscopy and molecular characterization of this population are included in this paper.

The species *Criconema demani* was described from a soil sample collected near the roots of *Carex* sp. in a meadow on the shore of the lake Tystrup Sø in Denmark (Micoletzky, 1925). Due to an inadequate original description, Raski and Riffle (1967) restudied the lectotype. This species has also been reported to be present in Estonia (Krall, 1965), the UK (Boag and Orton Williams, 1976), the Netherlands (Bongers, 1988), Belgium (Bert et al., 2003), Spain (Escuer and Bello, 1994), the Czech Republic (Háněl, 1996), Romania (Popovici and Ciobanu, 2000), Germany (Michiels and Traunspurger, 2004), the USA (Wehunt, Golden and Robbins, 1989), Venezuela (Crozzoli and Lamberti, 2002), Mexico (Luna-Guerrero et al., 2011), and Korea (Choi and Geraert, 1994).

In present times, it has been found near the roots of wild red raspberries (*Rubus idaeus* L.) in the Pechory district of the Pskov Region, Russia. The geographical location of the sampling site is 57° 48′ 27.18′′ N; 27° 38′ 1.536′′ E. It is the first record of this species in Russia.

## Materials and Methods

Nematodes were extracted from the soil samples using a modification of the decanting and sieving method (Flegg, 1967). For morphological studies, the nematodes were killed with hot water, fixed in 5 % formalin solution, and mounted in glycerin slides using the Seinhorst technique (Seinhorst, 1959).

For molecular studies, nematodes were fixed with 96% ethanol. Their total DNA was extracted using the K-Sorb kit (Syntol LLC, Russia) according to the manufacturer’s instructions. There were five replicates. Each replicate was a test tube with several nematode specimens. Two sets of primers were used for the amplification of the 18 S rRNA gene: the forward 18S39F (5′-AAA GAT TAA GCC ATG CAT G-3′) and the reverse 18S977R (5′-TTT ACG GTT AGA ACT AGG GCG G-3′), and the forward 18S900F (5′-AAG ACG GAC TAC AGC GAA AG-3′) and the reverse 18S1713R (5′-TCA CCT ACA GCT ACC TTG TTA CG-3′) (Olson et al., 2017). The forward D2A (5′-ACA AGT ACC GTG AGG GAA AGT TG-3′) and the reverse D3B (5′-TCG GAA GGA ACC AGC TAC TA-3′) (De Ley et al., 1999) primers were used for amplification of the D2–D3 expansion segments of the 28S rRNA gene. The partial cytochrome c oxidase subunit 1 gene was amplified with the forward primer COI-F5 (5′-AAT WTW GGT GTT GGA ACT TCT TGA AC-3′) and the reverse primer COI-R9 (5′-CTT AAA ACA TAA TGR AAA TGW GCW ACW ACA TAA TAA GTA TC-3′) (Powers et al., 2014). Amplifications were performed in a 2720 Programmable Thermal Cycler (Applied Biosystems, USA). PCR products were enzymatically purified using Exonuclease I (Thermo Scientific, USA) and shrimp alkaline phosphatase (SibEnzyme, Russia). Sequencing of PCR products was carried out with the same primers using genetic analyzer ‘ABI 3130xl’ (Applied Biosystems, USA). Low quality segments of sequences at the 5′ and 3′ ends were removed. Then, the newly obtained sequences were submitted to the GenBank database under accession numbers MH828123 and MH828124 (18S rRNA gene), MH828126 (28S rRNA gene), and MK248472 (COI gene).

## Results and Discussion

**Figure 1:**
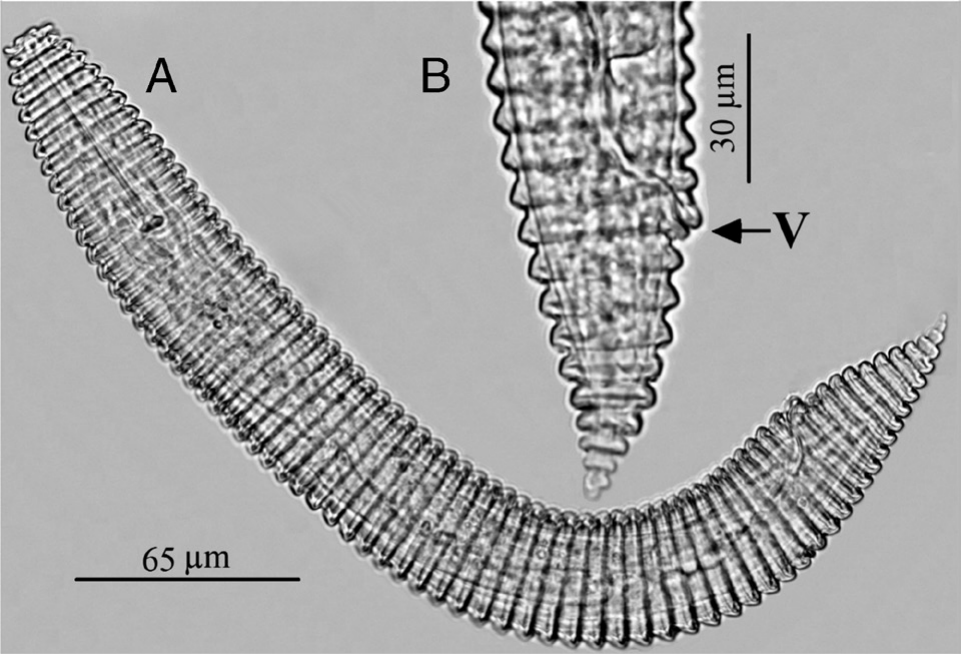
Light micrographs of *Criconema demani*. (A) Entire female and (B) Tail region.

Morphometrical characterization. Body is slightly curved ventrally. Annuli retrorse, margins smooth, no lateral differentiation. First and second annuli are equal in diameter. First annulus is usually slightly directed anteriorly; second annulus is not retrorse. The edges of annuli are smooth. Stylet is moderately long and robust, whereas cone is about 4/5 of total stylet length; knobs are 7 to 8 µm in diameter, slightly indented anteriorly, sometimes sloping posteriorly. Vulval lips are large and protruding; anterior lip covers the posterior. Spermatheca is small, round, and empty in all specimens. Tail is conical and tapers evenly, its terminal annuli are separated. Tail tip is usually straight; on some specimens, it is curved slightly dorsally, while on the others it is curved slightly ventrally. Anastomoses are very rare (Fig. 1 and Table 1).

**Table 1:** Measurements and ratios of 15 females of *Criconema demani* from the Pskov region of Russia, and their comparison with those from the literature. All measurements are in µm.

Morphological characters	This study	Brzeski (1998)	Crozzoli and Lamberti (2002)	Choi and Geraert (1994)
Body length	372.76 ± 37.5 (305–454)	380–530	331–386	381–437
Body width	31.9 ± 2.2 (28.7–35.5)	–	34–44	–
Esophagus length	98.91 ± 5.6 (85–109)	99–110	95–99	–
First annulus diameter	15.17 ± 1.1 (13.8–16.9)	–	13–14	–
Stylet length	64.9 ± 3.2 (60–68.8)	59–84	65–72	66.2–77.7
Tail length	32.2 ± 5.4 (25–42.9)	24–49	28–33	–
*Annule numbers*				
R	70.8 ± 2.3 (65–74)	63–77	62–66	61–68
Rst	13.9 ± 0.9 (12–16)	–	12–14	–
Roes	18.8 ± 1.1 (17–21)	17–20	17–19	–
Rex	20.9 ± 1.4 (19–23)	19–24	19–21	19–22
RV	11.7 ± 1.2 (10–13)	11–17	12–14	11–13
RVan	3.42 ± 0.5 (3–4)	3–5	3–4	4–5
Ran	8.04 ± 0.8 (7–9)	7–12	9–10	6–8
RB	5.3 ± 0.5 (4.5–6)	5–8	–	–
*Percentages*				
V	84.9 ± 1.2 (82.7–87)	84–88	84–86	84.1–87.2
St % L	17.2 ± 1.3 (15.5–19.7)	14–18	–	–
*Ratios*				
a	11.5 ± 1.4 (8.9–13.2)	9–13	8.8–10	10.2–11.7
b	3.8 ± 0.4 (3.3–4.6)	3.8–5	3.4–4	3.6–4.4
c	12.8 ± 1.9 (10.3–15.8)	9–16	11–13	12–16.5
VL/VB	1.71 ± 0.2 (1.4–2.0)	1.6–2.2	1.6–1.8	–

Molecular characterization. Despite the wide geographical distribution of the studied nematode species, as it is known from the literature, there are no molecular data for this species in the GenBank. There are two sequences of the species *Criconema longulum* (Gunhold, 1953) in the GenBank, and this species is most morphologically close to the species *C. demani*. One is a partial 18S rRNA gene sequence (KX344495), and the second is a partial cytochrome c oxidase subunit 1 gene sequence (MF770910).

The sequences of the 18S rRNA gene, the D2–D3 expansion segments of the 28S rRNA gene, and the COI gene obtained from different individuals in this study were identical to each other. 18S rRNA gene sequences of the studied specimens were identical by 94.7% with *C. longulum* from the USA (KX344495). The sequence of COI gene obtained in this study was identical by 95.7% to the sequence of the species *C. longulum* and shares less identity with another criconematid species deposited in GenBank.

The sequences of the D2–D3 expansion segments of the 28S rRNA gene were most similar to the *Criconema* sp. sequences from California, USA, with 90% similarity (FN433874, FN433864, FN433863, and FN433862). Other sequences of this segment of criconematid nematodes deposited in GenBank had lower than 90% similarity.
